# Supplementary material for: Population pharmacokinetics of benznidazole in neonates, infants and children using a new pediatric formulation
Source: PLoS Negl Trop Dis. 2023 May 31;17(5):e0010850. doi: 10.1371/journal.pntd.0010850 (PMC10259795; doi:10.1371/journal.pntd.0010850)
Supplement: S1 Trial Protocol — (PDF) [file pntd.0010850.s002.pdf]

CLINICAL TRIAL PROTOCOL

Population Pharmacokinetics Study of Benznidazole in Children with Chagas'Disease

**Name of product(s)/ Project code**	Benznidazole (N-benzil-2-nitro-1-imidazolacetamida)
**Drug Class**	Nitro-imidazole
**Phase**	Phase IV, population pharmacokinetics study
**Indication**	Chagas disease
**Protocol Number**	DNDi-CD-PEDBZ-001
**Sponsor**	DND*i*, Chemin Louis Dunant, 15, 1202 GENEVA Switzerland<br>Phone: +41 22 906 9230
**Co-sponsor**	Not applicable
**Coordinating Investigator/Principal Investigator**	Principal Investigator:<br>Jaime Altcheh<br>Hospital de Niños Ricardo Gutierrez, Buenos Aires, Argentina.<br><br>Co-Investigator:<br>Facundo Garcia-Bournissen,<br>Division of Clinical Pharmacology and Toxicology<br>Hospital for Sick Children, Toronto, Canada.<br><br>Other Co-Investigators to be defined.
**Protocol Synopsis Version / Date**	Version 2.0 of 12 October 2010.

The information contained in this document is confidential. It is to be used by investigators, potential investigators, consultants, or applicable independent ethics committees. It is understood that this information will not be disclosed to others without written authorisation from DNDi, except where required by applicable local laws

SIGNATURES

DNDi Signature Page

Medically qualified DNDi Officer

Signature ______________________ ______________________

Date of Signature (dd/mm/yy)

Name Isabela Ribeiro, MD
Title Senior Project Manager
Institution Drugs for Neglected Diseases initiative– DNDi
Address Regional Office - Latin America
Rua Santa Heloisa 5
22460-080 - Rio de Janeiro – Brazil

PK Expert

Signature ______________________ ______________________

Date of Signature (dd/mm/yy)

Name Facundo García Bournissen, MD
Title Clinical Fellow
Institution Division of Clinical Pharmacology & Toxicology
Hospital for Sick Children, University of Toronto
Address 555 University ave, 8th floor, room 8232
Toronto, ON M5G 1X8 Canada

Investigators Signature Page

I have read this protocol and agree that it contains all necessary details for carrying out this trial. I will conduct the trial as outlined herein and will complete the trial within the time designated.
I will provide copies of the protocol and all pertinent information to all individuals responsible to me who assist in the conduct of this trial. I will discuss this material with them to ensure they are fully informed regarding the drug and the conduct of the trial.
I will use only the informed consent form approved by the sponsor or its representative and will fulfill all responsibilities for submitting pertinent information to the Institutional Review Board/Independent Ethics Committee (IRB/IEC) responsible for this trial.
I agree that the sponsor or its representatives shall have access to any source documents from which case report form information may have been generated.

Coordinating Investigator

Investigator Signature ______________________ ______________________

Date of Signature (dd/mm/yy)

Name Jaime Altcheh
Title MD, Head of Parasitology Unit
Institution Hospital de Niños Ricardo Gutierrez
Address Gallo 1330 - 1425- Buenos Aires, Argentina

Principal Investigator at each trial site

Investigator Signature ______________________ ______________________

Date of Signature (dd/mm/yy)

Name
Title
Institution
Address

TABLE OF CONTENTS

TABLE OF CONTENTS 4
ABBREVIATIONS – GLOSSARY OF TERMS 7
PROTOCOL SYNOPSIS 8
1. BACKGROUND AND STUDY RATIONALE 14
2. STUDY OBJECTIVES AND ENDPOINTS 18
2.1.1. Primary Objective 18
2.1.2. Secondary Objectives 18
2.2. Study Endpoints 18
2.2.1. Primary Endpoint 18
2.2.2. Secondary Endpoint(s) 18
3. STUDY DESIGN AND STUDY DESIGN RATIONALE 19
3.1. Study design 19
3.2. Study duration and duration of subject participation 19
3.3. Rationale of study design 20
4. SELECTION OF SUBJECTS 21
4.1. Inclusion criteria 21
4.2. Exclusion criteria 22
The presence of any of the following will exclude a subject from study enrolment: 22
5. SCHEDULE OF EVENTS 22
6. ENROLMENT PROCEDURES 24
7. TREATMENTS 25
7.1. Investigational Product 25
7.2. Comparator standard treatment 25
7.3. Doses and treatment regimens 25
7.4. Drugs labelling, packaging 26
7.5. Accountability 26
7.6. Storage 27
7.7. Blinding and procedures for unblinding 27
7.8. Concomitant medications 27
8. STUDY ASSESSMENTS 27
8.1. Timing of Assessments 27
8.2. Baseline Assessments 28
8.3. Assessment of Efficacy 28
8.3.1. Assessments performed 29
8.4. Pharmacokinetics, Assessments*)* 29
8.5. Assessment of Safety 30
8.5.1. Laboratory examinations 30
8.6. Adverse event definitions and reporting 30
8.6.1. Adverse Event definition 30

8.6.2. Serious Adverse Event *31*
8.6.3. Eliciting Adverse Event information *31*
8.6.4. Adverse Event reporting period *32*
8.6.5. Adverse Event reporting requirements *32*
8.6.6. Grading of Adverse Event severity *32*
8.6.7. Adverse Event causality assessment *33*
8.6.8. Exposure in utero *34*
8.6.9. Adverse event follow up *34*

9. WITHDRAWAL CRITERIA 34

9.1. Rules for permanently interrupting study treatment 35

9.2. Subject withdrawal from the study and subject replacement 35

Subjects withdraw from the study will not be replaced ……………………………….35

10. DATA ANALYSIS AND STATISTICAL METHODS 35

10.1. Sample size determination 35

10.2. Definition of study populations included in the analysis 36

10.3. Subject Disposition 36

10.4. Baseline (optional) 36

10.5. Treatment Compliance (optional) 36

10.6. Efficacy Analysis 37

10.7. Safety Analysis 37

10.8. Analysis of other endpoints *(e.g. PK)* 38

11. DATA SAFETY MONITORING BOARD 38

12. QUALITY ASSURANCE AND QUALITY CONTROL PROCEDURES 39

12.1. Investigator's file 39

12.2. Case report forms (CRFs) 39

12.3. Source documents 39

12.4. Record Retention 39

12.5. Monitoring, audits and inspections 40

12.6. Audits and inspections 41

12.7. Data Management 41

12.8. Confidentiality of trial documents and subjects records 41

13. PROTOCOL AMENDMENTS 41

14. TERMINATION OF THE STUDY 42

15. ETHICS 42

15.1. Informed consent process 43

15.2. Ethical aspects of subject inclusion and study procedures 43

15.3. Ethical aspects of study treatments 43

15.4. Patient costs 43

16. INSURANCE AND LIABILITY 44

17. REPORTING AND PUBLICATION 44

18. REFERENCES 44

ABBREVIATIONS – GLOSSARY OF TERMS

AE	Adverse event
ALT	Alanine aminotransferase (SGPT)
ANMAT	Administración Nacional de Medicamentos, Alimentos y Tecnología Médica – Drug regulatory authority in Argentina.
AP	Alkaline Phosphatase
AST	Aspartate aminotransferase
AUC	Area under the curve
Bz	Benznidazole
CBC	Complete blood count
CD	Chagas Disease
CL	Clearance
Cmax	Maximum Concentration
Cmin	Minimum Concentration
CRF	Case Report Form
CTCAE	Common Terminology Criteria for Adverse Events
DNDi	Drugs for neglected diseases initiative
DSM	Data Safety Monitor
IEC	Independent ethics committee
FDA	Food and Drug Administration
GCP	Good clinical practice
ICH	International Conferences on Harmonization
Ka	Absorption rate constant
Ke	Elimination rate constant
LAFEPE	Laboratório Farmacêutico do Estado de Pernambuco
PCR	Polymerase Chain Reaction
PI	Principal investigator
PK	Pharmacokinetics
PO	Per os, oral administration
SAE	Serious adverse event
t1/2	Plasma terminal half-life (t=time)
ULN	Upper limit of normal
Vd	Volume of distribution
WBC	White blood cell
WHO	World Health Organization

PROTOCOL SYNOPSIS

**Protocol Title**	Population Pharmacokinetics Study of Benznidazole in Children with Chagas Disease
**Phase**	Population pharmacokinetics study
**Indication**	Chagas disease
**Protocol Number**	DNDi-CD-PEDBZ-001
**Background Information and Trial Rationale**	Treatment of Chagas disease (CD) has been always focused on pediatric population. Initially, treatment was recommended only to acute and congenital cases (including newborns diagnosed at birth), with good parasitological response of 60% to 85% of patients in the acute phase and more than 90% of congenitally infected infants treated in the first year of life. More recently, treatment recommendation has been extended for children with early chronic indeterminate form of disease up to 12y based on evidence published by mid to late 90's, indicating efficacy of ~60%, as assessed by seroconversion 3 to 4 years post-treatment.<br><br>The second report of WHO, Expert Committee (WHO, 2002) on Etiological treatment in the Chronic Phase stated that "given the epidemiological realities of each country, the consensus that people with Chronic Chagas Disease (individuals with positive serology for Chagas) should be treated with specific drugs has been established".<br><br>Despite existing treatment recommendations for children with CD (from birth to 12y), there is no formulation available that meets the needs of target pediatric population, especially the younger age groups.<br><br>Benznidazole (Bz), developed over 30 years ago and the main drug of choice, is only available in an 'adult' tablet strength of 100 mg (LAFEPE Benznidazol®). The dose recommended for children is 5-10 mg/Kg/day PO bid for 30-60 days, which has been extrapolated from adult patient data and empirically derived based on clinical experience.<br><br>With the lack of pediatric formulation, the 100mg tablet needs to be fractionated in ½ and ¼ tablets or prepared as extemporaneous formulations (macerated, diluted, suspension, etc) to adjust the dose to patient weight, often leading to sub or over-dosing, which may affect safety and efficacy of the treatment.<br><br>Furthermore, there have been no appropriate studies on the relationship of age to the pharmacokinetics (PK) properties and effects of Bz. In fact, only 2 studies on the PK of Bz in adults have been published to date, both in the 1970's.<br><br>Bz kinetics was first described in a PK study where 6 healthy female volunteers (22-24 years old) were exposed to a single 100mg tablet of Bz. Bz

PK fits a single compartment model, with C_{max} of 2.2 – 2.8 µg/ml observed at T_{max} 3–4 hs post-dosis. The estimated half life was 12 hs, and the volume of distribution 0.56 l/kg. In the second published study, 8 CD patients were treated with Bz 7mg/Kg/day PO for 30days, with plasma concentrations similar to those estimated based on the PK parameters obtained from the single-dose study. All patients reached steady-state within 10 days of treatment with plasma concentrations between 5.4 – 16.4 µg/ml.

With regards to children, there is an absolute lack of information on Bz PK in the pediatric population and its relationship with treatment safety and efficacy.

In order to respond to the need of a age-adapted, easy to use pediatric formulation, DNDi and LAFEPE have joined efforts to develop a 12.5 mg dispersible Bz tablet targeting treatment of CD in children < 20 Kg. Once this formulation is available, two pharmacokinetics studies are planned to be conducted: a comparative bioavailability study in adult healthy normal volunteers and a population pharmacokinetics study in young children.

The present protocol synopsis describes the population pharmacokinetics study of children with CD treated with benznidazole. The target population for this study will include 80 patients distributed over a broad age range: from newborns to children up to 12 years old, allowing for description of PK profiles for different age groups.

The group of newborns, from birth to - 2 years-old children, has been included as they represent the population of congenital cases. Current estimates of positive serology for CD in women at reproductive age vary considerably from country-to-country ranging from 5-40%, with vertical transmission rates of up to 12%. There is consensus that congenital infection may remain an important mode of transmission for another generation, and appropriate treatment targeting newborns is a possible control strategy (with very high chances of cure) with the new pediatric formulation.

Children 2-12 years-old have also been included as a target population, to represent those who may have been infected via congenital or vector-borne transmission, and usually present with the early chronic indeterminate form of the disease. In CD endemic countries this group of children are usually diagnosed through school or community screening programs, and also have a high chance of cure (> 60-75%) with Bz treatment.

Population PK has been chosen as the study design as it would minimise the number of samples per patient, an important requirement for studies conducted in the pediatric population. The dearth of PK data in adults and lack of information on the variability in the target population does not allow for power calculations and the use of optimal sampling design for definition of the timing of samples. Experts reviewed the available information and recommended sparse sampling, with 5 PK samples distributed over the absorption phase (1 sample), steady-state (2 samples) and elimination phase (2 samples). With a total of 5 PK measurements per patient and a total of 80 patients stratified by age, it is expected that PK curves and variability can be drawn with an adequate level of precision.

	PK data obtained from this study is expected to inform an age-adapted Bz regimen for the pediatric population affected by CD.
**Trial Objectives**	<u>Primary Objective:</u><br>To describe the population pharmacokinetics parameters of benznidazole in children with acute or early chronic indeterminate form of Chagas Disease.<br><br><u>Secondary Objectives:</u><br>• To evaluate if pharmacokinetics parameters are associated with parasitological cure (negative PCR) at the end of treatment<br>• To evaluate if benznidazole pharmacokinetics parameters are associated with the occurrence and severity of adverse events<br>• To evaluate the efficacy of benznidazole treatment at Day 60 (the end of treatment) through the assessment of parasitological cure by PCR.<br>• To evaluate the incidence of Serious Adverse Events, and/or adverse events leading to discontinuation of treatment in children
**Trial Endpoints**	Pharmacokinetics endpoints:<br>• Plasma level concentrations of benznidazol determined in children at first day of treatment (Day 0), steady state phase (D7 and Day 30) and at the end of treatment (Day 60)<br>• Population pharmacokinetics parameters of benznidazole in children, including CL, Vd, and Ka. Individual AUC, Cmax, Cmin, and t1/2 will be estimated using population parameters.<br><br>Efficacy endpoint:<br>• Parasitological cure rate as determined by qualitative PCR at the end of treatment (Day 60).<br><br>Safety endpoints:<br>• Rate of Serious Adverse Events and/or adverse events leading to treatment discontinuation<br>• Rate and severity of adverse events<br><br>Covariates to be evaluated: age, gender, weight, height, parasite load at baseline and phase of disease (acute vs chronic).
**Trial Design**	Open label, uncontrolled, single group assignment, stratified by age groups, population pharmacokinetics study.
**Main Entry Criteria**	A total of 80 patients with CD will be recruited for this study, including congenital cases, children with early chronic indeterminate form of

**Inclusion Exclusion**	disease as well as vector-borne acute cases.<br><br>Subject enrolment will be stratified by age: 40 patients in the group of newborns to 2 years (with a minimum of 10 newborns) and 40 patients in the group of > 2-12 years.<br><br>Patients must fulfil the following entry criteria to be eligible for enrolment in the study:<br><br><u>Inclusion Criteria</u>:<br><br>• Age between newborn (1day) – 12 years<br>• Diagnosis of *T. cruzi* infection by:<br>a. Direct microscopic examination or<br>b. Conventional serology, at least two positive tests (ELISA, IIF or HAI)<br>• Written informed consent form by parent/ legal representative<br>• Children assent if > 7 years<br><br><u>Exclusion Criteria</u>:<br><br>• Pre-term (< 37 weeks gestational age) or weight < 2500 g<br>• Female subject who has reached menarche<br>• Subjects presenting any other acute or chronic health conditions, that in the opinion of the PI, may interfere with the PK, efficacy and/or safety evaluation of the study drug<br>• Known history of hypersensitivity or serious adverse reactions to nitro-imidazoles<br>• History of CD treatment with benznidazole or nifurtimox in the past<br>• Immunocompromised patients (clinical history compatible with HIV infection, primary immunodeficiency or prolonged treatment with corticosteroids or other immunosuppressive drugs)<br>• Abnormal laboratory test values[1] at screening for the following parameters: total WBC count, platelet count, ALT, AST, total bilirubin and creatinine.<br>• Inability to comply with follow-up and/or not having a permanent address<br>• Any condition that prevents the subject from taking oral medication<br><u>*Note:*</u><br>*[1] Abnormal lab values will be fully described in the study protocol. Consider clinically significant or greater than CTC Grade 1 as a reference. Normal range for children may vary according to age.*<br>*Newborns with congenital Chagas Disease may present increased ALT/AST and bilirubin due to infection. For this group, ALT/AST and bilirubin will not be considered exclusion criteria unless considered clinically significant by the investigator.*

**Study Duration**	The total duration of subject participation in the study will be approximately 10 weeks.<br><br>Screening will occur within 2 weeks.<br><br>Once a subject is enrolled, he will have two follow-up visit during the treatment phase of the study (Day 7 ± 3 days, Day 30 ± 7 days), and one visit at the end of treatment (Day 60 ± 7 days).<br><br>Pharmacokinetics sampling will occur at Day 0 (at randomly selected time-point 2-5hs after first dose), at steady state phase [one sample to be collected at Day 7 and at Day 30 (at randomly selected time-points from pre-dose to 8hs post dose)], and at the end of treatment (two samples to be collected at randomly selected time-points 12 – 24hs after last dose at Day 60).<br><br>In addition, patients will be advised to return on any day during the follow-up period if they present any medical event.<br><br>Recruitment of the 80 patients is expected to occur within 6 months. The total study duration will be 15 months, from start-up to final study report.
**Test Drugs**	All 80 subjects recruited into the study will receive treatment with:<br>- Benznidazole (Laboratório Farmacêutico do Estado de Pernambuco -LAFEPE, tablet 12.5mg or 100mg), 7.5 mg/Kg/day PO (actual range of 5.5-8.5 mg/Kg/d), divided in two daily doses, for 60 days<br><br>Patients / care-taker / legal representative will be advised that treatment must be taken in two divided doses and with a meal.<br><br>At Day 0 and Day 30 visits the parent/legal representative will receive enough medication for the subsequent 30 days and a study form (diary) for daily recording of information regarding dose administration. Also, they must bring all remaining study drugs on Day 30 and Day 60 visits to check for treatment compliance and drug accountability.<br><br><u>Rescue treatment</u>:<br>Patients who do not present parasitological cure at the end of treatment (negative PCR) will receive nifurtimox 10-15mg/Kg/day, divided in two-three daily doses for 60 days.<br><br>Patients who do not tolerate the study treatment will be withdrawn from the study and will receive alternative treatment with nifurtimox 10-15mg/Kg/day, divided in two-three daily doses for 60 days.

**Statistics**<br>**Sample size**<br>**Randomisation**<br>**Summary of analysis**	No formal sample size determination has been performed. Sample size calculations for a given precision of the resulting PK parameter estimates require some prior knowledge regarding the variability in PK in the proposed study population.<br><br>With the lack of PK data of benznidazole in children, the sample size was defined based on the expert's discussions and previous experience with medications with simple PK, the minimum size for population pharmacokinetics evaluation and logistical considerations.<br>PK estimates drove the sample size calculations. With the anticipated proportion of clinical failures of 5%, a sample size of 18 patients would suffice to demonstrate the prevalence of failures with 10% precision and 95% confidence level.<br><br>An increased number of treatment discontinuations may occur in children with age >7 years-old. With an anticipated proportion of 10% clinical failures, a total of 35 children would be required to demonstrate the prevalence of failures with 10% precision and 95% confidence interval.<br><br>A minimum of 40 patients per age category was recommended by population PK experts to increase representativeness. Patient enrolment is to be stratified as follows:<br>- birth – 2 years: 40 patients to be enrolled, with a minimum of 10 newborns.<br>- 2 – 12 years: 40 patients.

1. Background and Study Rationale

Chagas disease (CD) ranks among the world's most neglected diseases. In Latin America, 21 countries are endemic for Chagas disease. Estimates from the 1980s indicated that some 16 million to 18 million individuals were infected (WHO, 1991). In the 1990s, after a series of multinational control initiatives, estimates of the number of infected people were revised to 9.8 million in 2001 (Schmunis, 2000). The estimated burden of disease in terms of disability-adjusted life years (DALYs) declined from 2.7 million in 1990 (World Bank, 1993) to 586,000 in 2001 (Mathers, C.D. *et al*. 2006). Recent estimates from PAHO (2006) indicate an overall prevalence of 7.54 million infected people and 55,185 new cases per year.

Chagas disease — also known as American trypanosomiasis— is a zoonotic disease caused by the protozoan hemoflagellate *Trypanosoma cruzi* that is mainly transmitted by large, blood-sucking, reduviid bugs of the subfamily *Triatominae*.

The continued migration from rural to urban areas in endemic countries resulted in large number of subjects infected with *T. cruzi* living in cities. The 'urbanization' of Chagas disease resulted in an increased risk of transmission in blood banks in Latin America countries (WHO, 2002), as well as in non-endemic areas (see below). Therefore, blood transfusion has been described as another route of transmission of Chagas disease, mainly in urban areas.

Transmission of Chagas disease to children occurs mainly by vectorial transmission, but also through congenital infection, in which mothers chronically infected with *T. cruzi* may transmit the disease to their offspring. The risk of congenital transmission seems to vary according to different epidemiological factors, such as the strain of the parasite, the parasitaemia of the mother, the existence of lesions in the placenta, and the geographical region (WHO, 2002).

Apart from vectorial, blood transfusion and congenital transmission, Chagas disease can also be transmitted by organ transplant as well as oral infection, classically described in the Amazon region and usually associated with ingestion of contaminated fruit juices such as 'açaí', sugar cane and others (Pereira *et al.*, 2009).

Starting in the 90's, the launch of programs for vector control such as the Southern Cone Initiative, and serological screening of blood banks has resulted in a great impact on the prevention of new cases caused by vectorial transmission and blood transfusion in the last decades. Despite the success of these campaigns, areas with infestation of triatomine bugs remain – and these are often associated with poverty and populations with poor health care assistance (Hotez *et al.*, 2008). It is estimated that 108 million people are still at risk of contracting the disease in Latin America (PAHO, 2006).

Beginning in the 1960s and continuing to the present, migration from endemic countries of Latin America to the United States, Canada, Europe, Japan and Australia resulted in the diagnosis of Chagas disease in non-endemic countries and the worldwide spread of the disease (Schmunis, 2007). In these regions, the disease can occur through blood transfusion, organ transplantation and congenital infection. In the US only, it has been estimated that 166-638 newborns were infected with *T. cruzi* in 2000, taking into account the number of women in reproductive age migrating to the US and the estimated prevalence of *T. cruzi* infection in their country of origin (Yadón & Schmunis, 2009).

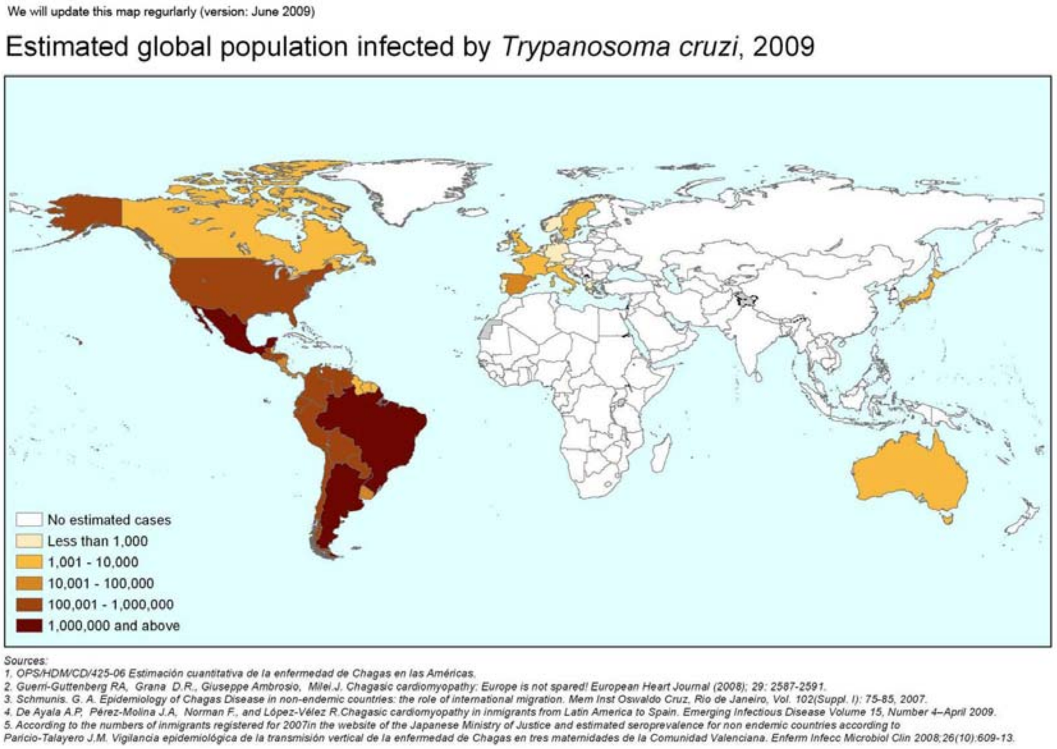

Source: World Health Organization, 2009

Clinically, human Chagas disease has two phases, acute and chronic. The acute phase, which lasts a few weeks, is a febrile and toxemic illness, during which the parasite can be detected by direct microscopic examination of fresh blood (Pinto-Dias, 2006). In the chronic phase, the diagnosis depends on hemocultures of varying degree of sensitivities, xenodiagnosis , PCR or detecting IgG antibodies (Rodrigues Coura & Castro, 2002). Untreated, the chronic phase continues for the rest of a person's life. It begins with no specific symptoms or clinical manifestations for a period of approximately 10 or 15 years, the "indeterminate form" of the disease (Pinto-Dias, 2006). About 20 to 50% of chronic Chagas patients over the ensuing years, depending on the endemic area analyzed, will develop involvement of the heart or gastrointestinal tract (Rodrigues Coura & Castro, 2002).

Current therapy for Chagas disease is limited to two nitroheterocyclic drugs, Nifurtimox and Benznidazole, the later being most widely used drug for Chagas disease treatment.

Benznidazole (Bz) is a nitroimidazole which was introduced by Roche in 1971 as Rochagan (Brazil) and Radanil (Argentina and other Spanish-speaking countries), and it is currently produced by 'Laboratório Farmacêutico do Estado de Pernambuco' (LAFEPE), Brazil. Its mechanism of action is generation of oxidative or reductive stress due to the production of reduced nitro radicals, which in turn leads to inhibition of protein and DNA synthesis. It is supplied in tablets with 100mg of drug substance and administered twice daily at a dose 5 mg/kg body weight/day for adults and 5-10 mg/kg body weight/day for children for 60 days. Most common adverse effects for Bz

include: allergic dermopathy, peripheral sensitive neuropathy, generalized oedema, fever, leukopaenia and articular and muscular pain.

Treatment of Chagas disease has been always focused on pediatric population. Initially, treatment was recommended only to acute and congenital cases (including newborns diagnosed at birth), with good parasitological response of 60% to 85% of patients in the acute phase (Kirchhoff LV, 2003) and more than 90% of congenitally infected infants treated in the first year of life (Rodrigues Coura & Castro, 2002). More recently, treatment recommendation has been extended for children with early chronic indeterminate form of disease up to 12-14 years based on evidence published by mid to late 90’s, indicating efficacy of ~60%, as assessed by seroconversion 3 to 4 years post-treatment (de Andrade *et al.*, 1996, Sosa-Estani *et al.*, 1998). Late chronic phase is treated at the clinician’s discretion.

In 2002, the second report of WHO, Expert Committee (WHO, 2002) on Etiological treatment in the Chronic Phase stated that “given the epidemiological realities of each country, the consensus that people with Chronic Chagas Disease (individuals with positive serology for Chagas) should be treated with specific drugs has been established”.

Despite existing treatment recommendations for children with CD (from birth to 12y), there is no formulation available that meets the needs of target pediatric population, especially the young age groups.

Benznidazole, developed over 30yrs ago and the main drug of choice, is only available in an ‘adult’ tablet strength of 100mg (LAFEPE Benznidazol®). The dose recommended for children, 5-10mg/Kg/day PO bid for 30-60 days, has been extrapolated from adult patient data and empirically derived based on clinical experience.

With the lack of pediatric formulation, the 100mg tablet needs to be fractionated in ½ and ¼ tablets or prepared as extemporaneous formulations (macerated, diluted, suspension, etc) to adjust to patient weight, which often leads to sub or over-dosing, that may affect safety and efficacy of the treatment (Ribeiro *et al.*, 2009).

Furthermore, there have been no appropriate studies on the relationship of age to the pharmacokinetic (PK) properties and effects of benznidazole. In fact, only 2 studies on the PK of benznidazole in adults have been published to date, both in the 1970’s.

Benznidazole kinetics was first described in a PK study where 6 female volunteers (22-24y) were exposed to a single 100mg tablet of Bz. PK fits a single compartment model, with C_{max} of 2.2 – 2.8 µg/ml observed at T_{max} 3–4 hs post-dosis. The estimated half life was 12 hs, and the volume of distribution 0.56 l/kg (Raaflaub J & Ziegler WH, 1979). In the second published study, 8 patients were treated with Bz 7mg/Kg/day PO for 30days, with plasma concentrations similar to those estimated based on the pharmacokinetic parameters obtained from the single-dose experiment. All patients reached steady-state within 10 days of treatment with plasma concentrations between 5.4 – 16.4 µg/ml (Raaflaub J., 1980).

With regards to children, there is an absolute lack of information on Benznidazole PK data for pediatric population and its relationship with treatment safety and efficacy (Garcia-Bournissen *et al.*, 2009). Information on PK of Benznidazle for children is

urgently needed.

In order to respond to the need of a age-adapted, easy to use pediatric formulation, DNDi and LAFEPE have joined efforts to develop a 12.5mg dispersible benznidazole tablet which is targeting treatment of CD children < 20 Kg. Once this formulation is available, two pharmacokinetics studies are planned to be conducted: a comparative bioavailability study in adult healthy normal volunteers and a population pharmacokinetics study in young children.

The present protocol is a population pharmacokinetics study of children with CD treated with benznidazole. The target population for this study will include 80 patients distributed over a broad age range: from newborns to children up to 12 years old, allowing for description of PK profile for different age groups.

Newborns-2 years-old children have been included as they represent the population of congenital cases. Current estimates of positive serology for CD in women at reproductive age vary considerably from country-to-country ranging from <5% in countries with successful vector control programs (Brazil, Chile and Uruguay), to higher rates of >5-20% in other countries (Argentina, Bolivia, others), but acknowledging heterogeneity within countries, with prevalence of up to 40% at community levels (Carlier & Torrico, 2003; Yadón & Schmunis, 2009). The transmission rate of congenital *T. cruzi* infection (number of congenital cases/number of chagasic mothers) in the Southern Cone countries also varies widely, from 1% in Brazil to 4 to 12% Argentina, Bolivia, Chile (Carlier & Torrico, 2003). There is consensus that congenital infection may remain an important mode of transmission for another generation, and appropriate treatment targeting newborns is a possible control strategy (with very high chances of cure) with the new pediatric formulation.

Children 2-12 years-old have also been included as the target population, to represent those who may have been infected via congenital or vector-borne transmission, and usually present with the early chronic indeterminate form of the disease. In CD endemic countries this group of children are usually diagnosed through school or community screening programs, and also have a reasonable chance of cure (>60-75%) after Bz treatment (Andrade, 2004 and Sosa-Estani, 2006).

Population pharmacokinetics has been chosen as the study design in order to minimise the number of samples in the pediatric population (FDA, 1998 and FDA, 1999).

The dearth of PK in adults and lack of information on the variability in the target population does not allow for power calculations and the use of optimal sampling design for definition of the timing of samples. Experts reviewed the available information and recommended sparse sampling, with a total of 5 PK samples to be collected during the absorption phase (1 sample), steady-state (2 samples) and elimination phase (2 samples). All PK samples will be 'micro-samples' of 100μL collected in filter-paper. With a total of 5 PK measurements per patient and a total of 80 patients stratified by age, it is expected that PK curves and variability can be drawn with an adequate level of precision.

PK data obtained from this study is expected to inform an age-adapted benznidazole regimen for pediatric population affected by CD.

2. Study Objectives and Endpoints

2.1. Objectives

2.1.1. Primary Objective

To describe the population pharmacokinetic parameters of benznidazole in children with acute or early chronic indeterminate form of Chagas Disease.

2.1.2. Secondary Objectives

- To evaluate if pharmacokinetics parameters are associated with parasitological cure (negative PCR) at the end of treatment (EOT).
- To evaluate if benznidazole pharmacokinetic parameters are associated with the occurrence and severity of adverse events.
- To evaluate the efficacy of benznidazole treatment at Day 60 (the end of treatment) through the assessment of parasitological cure by PCR.
- To evaluate the incidence of Serious Adverse Events, and/or adverse events leading to discontinuation of treatment in children.

2.2. Study Endpoints

2.2.1. Primary Endpoints

Pharmacokinetics Endpoints

- Plasma level concentrations of benznidazol determined in children at first day of treatment (Day 0), steady state phase (D7 and D30) and at the end of treatment (Day 60).
- Population pharmacokinetic parameters of benznidazole in children including CL, Vd, and Ka. Individual AUC, Cmax, Cmin, and t1/2 will be estimated using population parameters.

2.2.2. Secondary Endpoints

Efficacy Endpoints

- Parasitological cure rate as determined by qualitative PCR at the end of treatment (Day 60)

Safety endpoints:

- Rate of Serious of Adverse Events and/or adverse events leading to treatment discontinuation
- Rate and severity of adverse events

Covariates to be evaluated: age, gender, weight, height, parasite load at baseline and phase of disease (acute vs chronic).

3. Study design and study design rationale

3.1. Study design

The present study is an open label, uncontrolled, single group assignment, stratified by age groups, population pharmacokinetics study.

As the main objective of this study is to describe the pharmacokinetics of Bz in children with Chagas disease, this is a single treatment study, with no comparator. Nevertheless, the efficacy of Bz treatment will be described at the end of treatment, as well as the relationship of pharmacokinetics and pharmacodynamics.

Recruitment will occur in 5 sites, with one back-up site to be initiated depending on recruitment rates at the 2 initial months. Sites are located in Buenos Aires and in CD endemic areas in the North of Argentina (Jujuy, Salta and Santiago del Estero). The inclusion of study sites in endemic areas allow for active patient screening and access to congenital cases diagnosed at birth as well as vector-borne acute cases.

The 2 sites located in Buenos Aires are reference centers for treatment of CD in the capital. The majority of cases are migrants from endemic areas (including neighboring countries such as Bolivia and Paraguay) and mother-to-child infections. Newborn and all siblings are screened for CD from all mothers with positive serology identified during pre-natal care.

Recommendations of Bz treatment for Chagas disease in children is 5-10mg/Kg/day for 30-60 days (WHO, 2002). In this trial, the target dose of 7.5mg/Kg/day PO BID for 60 days will be used to avoid too much variability on drug regimen and adequate PK determination. Tables with the precise amount of Bz to be administered based on patient weight (number of 12.5mg pediatric tablet or 100mg tablet) will be provided to Investigators, to ensure that the same treatment is provided at the different study sites.

3.2. Study duration and duration of subject participation

The subject participation in the study will be approximately 10 weeks, considering 2 weeks for screening and the 60 days treatment with Bz. The last study visit is at the end of Bz treatment, and no further follow-up is expected for this PK study.

The choice for 60 days treatment was based on recommendations (WHO, 2002; Ministerio de Salud de la Nación, Argentina, 2005) and it is in line with current practice at study sites.

Once a subject is enrolled, he will have two follow-up visits during the treatment

phase of the study (Day 7 ± 3 days, Day 30 ± 7 days), and one visit at the end of treatment (Day 60 ± 7 days). Patients will be followed for a longer period of time as part of their routine management; however these assessments are not considered part of the study.

Pharmacokinetics sampling will occur at Day 0 (at randomly selected time-point 2-5hs after dosing), at steady state phase [one sample to be collected at Day 7 and one sample at Day 30 (at randomly selected time-points from pre-dose to 8hs post dose)], and at the end of treatment (two samples to be collected at a randomly selected time-points 12-24hs after last dose at Day 60).

In addition, patients will be advised to return on any day during the follow-up period if they present any medical occurrence or adverse events. Sampling for pharmacokinetics will be obtained during these visits, only if blood draws are done as part of patient's routine case management for investigation of medical occurrence.

Recruitment of the 80 patients is expected to occur within 6 months. Therefore, the timeline of First Subject In (FSI) and Last Subject Out (LSO) is 8 months. However, the total study duration is estimated to be 15 months, from start-up phase to final study report.

3.3. Rationale of study design

The target population of the study is children, from newborns to 12 years old, due to the absolute lack of PK information for this age group, which has been treated over the last 30-40 years with Bz with a treatment regimen that has been extrapolated from adult data and based on experience and consensus.

Stratification by age in this trial is necessary due to expected differences in pharmacokinetics profile of newborn-2y old and children > 2-12 years old. As it is expected that recruitment of newborns might be more difficult than other age groups, it has been established that a minimum of ten (10) newborns shall be recruited in the first age stratum.

Population pharmacokinetics have been chosen as the study design allows sparse sampling from the patients recruited for the study, especially considering the limitation in blood collections and volume when dealing with pediatric population. A micro-sample method for PK analysis has been developed in order to allow for very low blood volume collection.

The dearth of PK in adults and lack of information on the variability in the target population does not allow for power calculations and the use of optimal sampling design for definition of the timing of the PK samples. Experts reviewed the available information and recommended sparse sampling, with 5 PK samples distributed over the absorption phase (1 sample), steady-state (2 samples) and elimination phase (2 samples). With a total of 5 PK measurements per patient and a total of 80 patients stratified by age, it is expected that PK curves and variability can be drawn with an adequate level of precision.

Figure 1- Overall study design

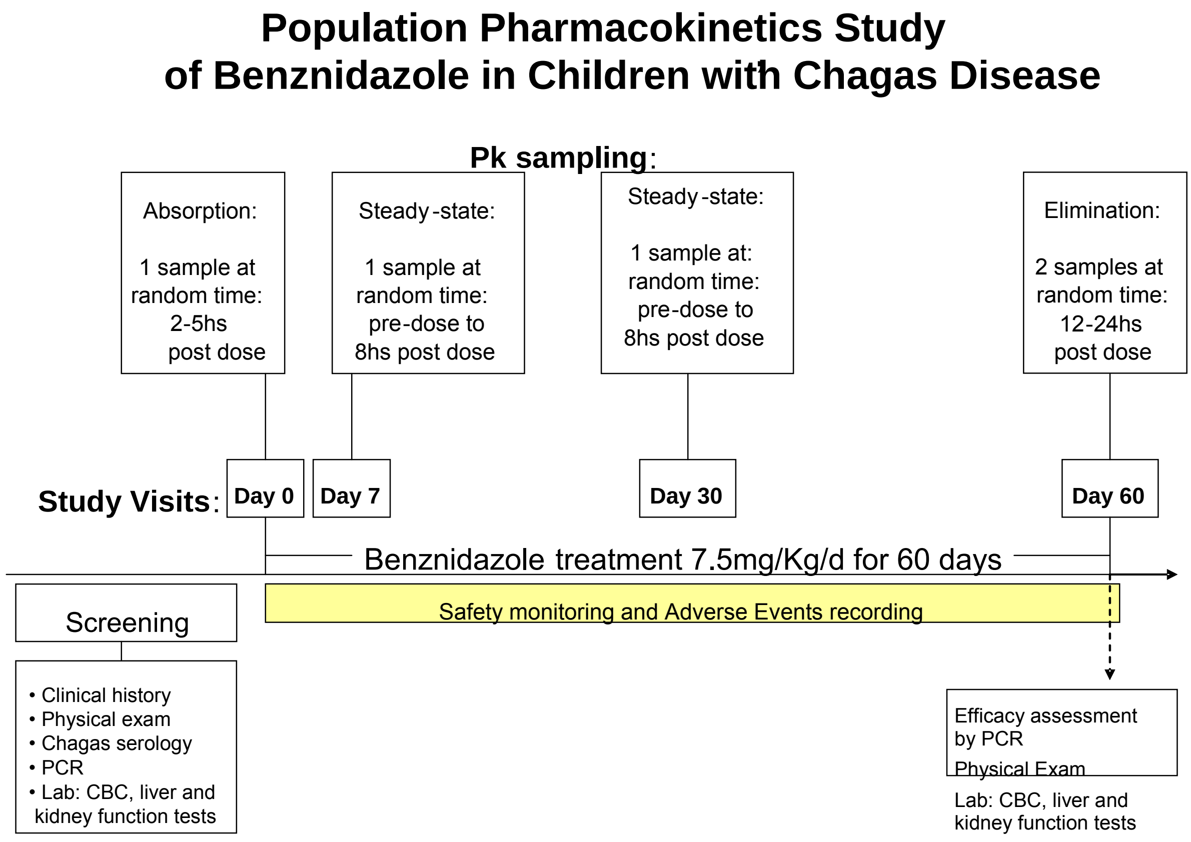

4. Selection of Subjects

A total of 80 patients with CD will be recruited for this study, including congenital cases, children with early chronic indeterminate form of disease as well as vector-borne acute cases.

Subject enrolment will be stratified by age: 40 patients in the group of newborns to 2 years (with a minimum of 10 newborns) and 40 patients in the group of > 2-12 years.

The following eligibility criteria were designed to select subjects for whom the protocol treatment is considered appropriate. All relevant medical and non-medical conditions should be taken into consideration when deciding whether this protocol is suitable for a particular subject. Eligibility criteria may not be waived by the investigator. Any questions regarding a subject's eligibility should be discussed with DNDi's medically qualified trial manager prior to a subject's enrollment.

4.1. Inclusion criteria

Subjects must meet **all** of the following inclusion criteria to be eligible for enrollment into the study:

- Age between newborn (1day) – 12 years
- Diagnosis of *T. cruzi* infection by:
    a. Direct microscopic examination or
    b. Conventional serology, at least two positive tests (ELISA, IIF or HAI)
- Written informed consent form by parent/ legal representative
- Children assent if > 7 years

4.2. Exclusion criteria

The presence of any of the following will exclude a subject from study enrolment:

- Pre-term (< 37 weeks gestational age) or weight < 2500 g
- Female subject who has reached menarche
- Subjects presenting any other acute or chronic health conditions, that in the opinion of the PI, may interfere with the PK, efficacy and/or safety evaluation of the study drug
- Known history of hypersensitivity or serious adverse reactions to nitro-imidazoles
- History of CD treatment with benznidazole or nifurtimox in the past
- Immunocompromised patients (clinical history compatible with HIV infection, primary immunodeficiency or prolonged treatment with corticosteroids or other immunosuppressive drugs)
- Abnormal laboratory test values at screening for the following parameters: total WBC count, platelet count, ALT, AST, total bilirubin and creatinine. Exception for this criterion is considered for newborns with congenital Chagas Disease, for whom ALT/AST and bilirubin will not be considered exclusion criteria unless considered clinically significant by the investigator.
- Inability to comply with follow-up and/or not having a permanent address
- Any condition that prevents the subject from taking oral medication

5. Schedule of events

The schedule of events for this trial is described in the table below:

Table 1. Study assessments to be completed during the study visits:

Schedule of Events					
	**Screening**	**Treatment period**			**End of treatment**
**Protocol activities and forms to be completed**	-D14 to -D1	Day 0	Day 7 (-1/+3 days)	Day 30 (± 7 days)	Day 60 (± 7 days)
Informed Consent Form	X				
Complete Medical History	X				
Demographics and history of medications	X				
Complete physical exam, including weight/height	X				
Vital Signs[1]	X	X	X	X	X
Simplified Physical Exam and Clinical assessment [2]		X	X	X	X
Chagas disease diagnosis:<br>- children < 8mo: direct microscopic exam[3]<br>- children ≥ 8mo: positive CD serology[4]	X				
PCR[5]	X				X
Hematology[6]	X		X	X	X
Chemistry[7]	X		X	X	X
Adverse Events		X (post-dose)	X	X	X
Concomitant Medications		X	X	X	X
PK sample(s)[8]		X	X	X	X

1. Vital signs to be assessed are body temperature, heart and respiratory rate. During the treatment period vital signs will limit to body temperature and heart rate.

2. Simplified physical exam aims to evaluate adverse events;

3. Children < 8 months and suspected acute CD cases: direct microscopic examination will be performed. One mL (1mL) of heparinized blood will be collected, with the examination of buffy coat in 2-3 slides. The patient must have a positive diagnosis to be eligible for the study.

4. Children > 8months: a 2mL blood sample will be collected at screening for conventional CD serology. Subject must have at least 2 positive tests (ELISA, IIF or HAI) to be eligible.

5. PCR will be performed in a 2 ml blood sample, which is to be mixed with one volume of 2 X lysis buffer containing 6 M guanidine hydrochloride) and 200 mM EDTA, pH 8.0

6. Hematology lab parameters will include: haemoglobin, total WBC count, differential WBC count, platelets count.

7. Chemistries lab parameters will include: ALT, AST, total, direct and indirect bilirubin, alkaline phosphatase and creatinine.

8. Sampling for pharmacokinetics:
    - Blood samples will be collected from each subject at the following time-points (all PK samples will be micro-samples of approximately 100µL in filter paper*):
        - Day 0, after administration of 1st dose, at a randomly selected time between 2-5hs post dose
        - Steady-state samples:
            - Day 7: one sample to be collected at a random time-point between pre-dose and 8hs post dose
            - Day 30: same as for Day 7.
        - End of treatment (Day 60): two samples to be collected at random time-points between 12-24hs post last dose

    The total blood volume to be collected for the 5 PK samples will be 0.5 mL.

    Furthermore, if a patient comes for an unscheduled visit due to an AE, and there is a need for blood collection for safety evaluation, a PK micro-sample of approximately 100µL will also be collected in a filter paper.

    *Analytical methods to allow micro-sampling are under development at 'Núcleo de Desenvolvimento Farmacêutico e Cosméticos' (NUDFAC, Recife, Brazil). Procedures for specimen collection will be updated in the study manual of operations.

ECG assessment will not be required per protocol. The exam will be requested at discretion of the responsible medical staff.

6. Enrolment procedures

Potential subjects will be identified at or referred to the CD treatment centres in different study sites; followed by appropriate screening and enrollment procedures including clinical assessments, CD diagnosis (serology or direct test for congenital cases), laboratory assessments for heamatology, biochemistry and PCR. If the subject is found to be eligible for the trial he/she will be invited to participate in the study through the informed consent process. In order for the subject to be enrolled in the study the informed consent form (and the assent form, if applicable) must be obtained from the parents/guardian by the Principal Investigator or the delegated study physician prior to any study specific procedure (e.g. PCR sample if not routine should be obtained only after full consent is obtained).

All sites participating in this trial should complete a Subject Screening/Enrolment Log to reflect screen date, screening number, referral source, screening status, date when informed consent was obtained, date of enrolment, study enrolment number and reason for not enrolling, whenever applicable.

Enrolment and screening procedures should occur up to two weeks or less (i.e. –D14 to –D1) from the intended initiation of study therapy. Enrolment procedures as well as confirmation of enrolment should be retained in the subject's source documentation.

This is a single arm, open label study; therefore, all subjects enrolled will receive study medication. Patients will be assigned, in ascending order, a study identification number according to the site number and order recruited. Each patient will be given an individually numbered treatment pack which contains sufficient tablets for the full course of therapy plus two extra tablets per day in case the patient vomits.

Envelopes with the allocated schedule of randomly selected times for sampling for the population pharmacokinetics analysis will be open at the time of assignment of the study identification number.

7. Treatments

7.1. Investigational Product

Benznidazole (N-benzyl-2-nitro- 1-imidazolacetamide) is a nitro-imidazole compound that was registered by Roche in 1971 and is now licensed to Laboratório Farmacêutico do Estado de Pernambuco S/A – LAFEPE, Brazil. Its mechanism of action is generation of oxidative or reductive stress due to the production of reduced nitro radicals, which in turn leads to inhibition of protein and DNA synthesis.

Benznidazole will be provided by LAFEPE as 12.5mg dispersible pediatric tablet and 100mg adult tablet. Both formulations will be provided in blisters with 10 tablets.

7.2. Comparator standard treatment

This is a single assignment study, in which all patients will be treated with Benznidazole.

7.3. Doses and treatment regimens

All 80 subjects recruited into the study will be treated with Benznidazole (Laboratório do Estado de Pernambuco -LAFEPE, tablet 12.5mg or 100mg), 7.5 mg/Kg/day PO, divided in two daily doses, for 60 days.

Patients $\geq$ 14Kg will be treated with benznidazole 12.5mg tablet (maximum of 4 tablets PO BID), and patients > 14kg will be treated with 100mg adult tablets. A table of number of tablets per weight will be used by Investigators in order to allow for same dosing across study sites, and to be as close as possible to the target dose of 7.5mg/Kg/day. As the tablets are scored, it will be possible to adjust the dose to ½ tablets, if needed.

Patients/caretaker/legal representative will be instructed on the exact dosing to be administered to the child, and will be advised that treatment must be taken in two divided doses and with a meal.

For young children to be treated with pediatric dispersible tablet, the patient parent/caretaker/guardian will be instructed to put the tablet into a spoon with a liquid solution, and after dispersion the liquid can be administered to the child.

At Day 0 and Day 30 visits, the parent/legal representative will receive enough medication until the next scheduled visit and a form (diary) for daily recording of information regarding dose administration. Also, they must bring all remaining study drugs on Day 30 and Day 60 visits to check for drug accountability.

Rescue treatment:

Patients who do not present parasitological cure at the end of treatment (negative PCR) will receive nifurtimox 10-15mg/Kg/day, divided in two-three daily doses for 60 days.

Patients who do not tolerate the study treatment will be withdrawn from the study and will receive alternative treatment with nifurtimox 10-15mg/Kg/day, divided in two-three daily doses for 60 days.

7.4. Drugs labelling, packaging

The adults' tablets of 100mg and 12.5mg pediatric tablets will be provided by LAFEPE.

Benznidazole labels are shown below:

Benznidazole 12.5 mg tablet label	Benznidazole 100 mg tablet label
Benznidazole 12.5 mg<br>Contains 10 tablets for oral administration<br><br>Weight < 14 Kg Patient No:<br><br>Dosage: 7.5mg/Kg/day x 60 days<br>Please place all unused drugs in sachet for drug accountability<br><br>For clinical trial use only<br>Keep out of the reach of children<br>Not for sale / No commercial value<br><br>Principal Investigato of<br>the study site:: ___________________<br><br>Manufacturer: LAFEPE, Brazil<br>Sponsor: Drugs for Neglected Diseases initiative (DNDi)<br><br>Batch Number:xxxxx Expiry Date: xx-xx-xx<br>Storage conditions: Room temperature (15-30°C). Do not refrigerate or freeze.	Benznidazole 100 mg<br>Contains 10 tablets for oral administration<br><br>Weight > 14 Kg Patient No: ___________<br><br>Dosage: 7.5mg/Kg/day x 60 days<br>Please place all unused drugs in sachet for drug accountability<br><br>For clinical trial use only<br>Keep out of the reach of children<br>Not for sale / No commercial value<br><br>Principal Investigator of<br>The study site: ___________________<br><br>Manufacturer: LAFEPE, Brazil<br>Sponsor: Drugs for Neglected Diseases initiative (DNDi)<br><br>Batch Number:xxxxx Expiry Date: xx-xx-xx<br>Storage conditions: Room temperature (15-30°C). Do not refrigerate or freeze.

7.5. Accountability

All Investigational Product will be shipped from LAFEPE to the main site, Hospital de Niños Ricardo Gutierrez, in Buenos Aires. Dr. Altcheh will distribute the study medication to the other study sites.who will be responsible for drug accountability

Study specific forms will be used for drug accountability. Adequate records on Benznidazole receipt, use, return, loss, or other disposition will be documented and maintained by the study site investigators and supervised by the PI. Study monitors will be also in charge of checking for drug accountability during the regular monitoring visits to the study sites.

All study medications must be kept in a locked room at each of the study sites, with restricted access only by the pharmacist or the study investigators. Benznidazole that will be provided for this study must not be used for other purposes other than this protocol. Under no circumstances the investigator or site staff may supply the study medication to other investigators or health care services, or allow the medication to be used other than as directed by this protocol without prior authorization from DNDi.

7.6. Storage

Benznidazole tablets are stable at room temperature (15 – 30° C), and do not need to be shipped or stored under refrigeration. However, if local temperatures are above 30 ^{o}C, benznidazole should be kept in a room with air conditioning. A temperature log with regular recordings of temperature in the storage room will be available.

7.7. Blinding and procedures for unblinding

Blinding does not apply for this opened, single medication assignment study.

7.8. Concomitant medications

Subjects may receive concomitant therapy for medical occurrences during the course of the study.

All concomitant medications taken by the patient during the study, from the date of signature of the informed consent, will be recorded in the appropriate section of the Case Record Form.

8. Study Assessments

8.1. Timing of Assessments

As described in Table 1, study assessments will be done at Screening (Days -D14 to -D1), Day 0 (treatment onset), Day 7 (-1/+3 days), Day 30 (± 7 days) and Day 60 (± 7 days).

Efficacy assessment will be done at Day 60, at the end of treatment.

Pharmacokinetics assessments will be done at Day 0 (absorption phase), Day 7 and Day 30 (steady-state phase), Day 60 (elimination phase), and at any unscheduled visit that requires blood sample collection. Safety assessments will be done at Day 0 (post-dose), Day 7, Day 30, Day 60, and at any unscheduled visit.

8.2. Screening and Baseline Assessments

During screening, the following assessments will be done in order to evaluate patient eligibility for the study:

- Complete medical history with an emphasis on Chagas Disease
- Demographic data and history of medications
- Physical examination, body weight and height, vital signs, and body temperature.
- Microscopic direct examination for children < 8 months: one mL (1mL) of heparinized blood will be collected, with the examination of buffy coat in 2-3 slides. The patient must have a positive diagnosis to be eligible for the study.
- Chagas Disease serology for children ≥ 8months: a 2mL blood sample will be collected at screening for conventional CD serology. Subject must have at least 2 positive tests (ELISA, IIF or HAI) to be eligible.
- PCR (1 blood sample of 2 mL) for all children
- Clinical safety laboratory evaluations: haemoglobin, total WBC count, differential WBC count, platelet count, ALT, AST, total, direct and indirect bilirubin, alkaline phosphatase and creatinine.

  Although all these lab parameters will be measured, the ones to be considered for patient eligibility are: total WBC count, platelet count, ALT, AST, total bilirubin and creatinine.

  As newborns with congenital Chagas Disease may present increased ALT/AST and bilirubin due to infection, these lab parameters will not be considered exclusion criteria for this group unless considered clinically significant by the investigator.

At baseline visit the patient will have a clinical assessment and physical exam including vital signs before the first dose of medication is administered. Concomitant medications will be recorded in the appropriate CRF.

Patient will receive the first dose of medication at the clinic and will be observed for occurrence of adverse events until the timing for the PK sampling. If any AE occurs, the patient will receive medical assistance, and the event will be recorded in the appropriate CRF.

A blood sample of 100µL will be collected for PK analysis at a randomly selected time point between 2-5hs post-dose.

8.3. Assessment of Efficacy

Efficacy of study treatment will be assessed by PCR at the end of treatment (Day 60). PCR will not be considered an inclusion criterion, and it is expected that 5-10% of patients will be PCR negative at screening. These patients shall not be included in this analysis.

8.3.1. Assessments performed

A blood sample of 2mL in will be collected at screening and at Day 60 for PCR analysis. Immediately, the blood drawn will be added to a tube containing one volume (2mL) of a solution of Guanidine/ClH 6M EDTA 0.2M pH 8,0 Buffer (GEB) (Shijman, 2003). Samples shall then be stored in a refrigerator.

All PCR samples will be shipped to Dr. Alejandro Schijman at CONICET, Laboratorio de Biologia Molecular de la Enfermedad de Chagas, Buenos Aires, Argentina, where PCR assays will be performed.

The PCR technique to be employed will be the one described on the Workshop & Symposium on 'Standardization and validation of the clinical use of PCR for the detection of *T. cruzi* infection", coordinated by Dr. Schijman.

A patient will be considered cured (parasitological cure) if a PCR that was positive at screening turned to be negative at Day 60.

8.4. Assessment of Pharmacokinetics

Plasma level concentrations of benznidazole will be determined during the absorption, steady-state and elimination pharmacokinetics phases.

The PK parameters to be modelled are CL, Vd and Ka (although this will depend on the final compartmental model chosen). AUC, Cmax, Cmin, and T1/2 cannot be directly measured individually in a population pharmacokinetics modelling approach with sparse sampling, but they will be estimated on the basis of the model – generated parameters.

Blood samples will be collected from each subject at the following time-points (one sample of approximately 100µL*/timepoint:

- Day 0, after administration of 1st dose, at a randomly selected time-point between 2-5hs post dose
- Steady-state samples:
  - Day 7: one sample to be collected at a random time-point between pre-dose and 8hs post dose
  - Day 30: same as for Day 7.
- End of treatment (Day 60): two samples to be collected at random time-points between 12-24hs post last dose.

Furthermore, if a patient comes for an unscheduled visit due to an AE, and there is a need for blood collection for safety evaluation, a PK micro-sample of 100µL will also be collected in a filter paper.

The time of drug administration and blood draws will be recorded in the appropriate sections of the CRF.

** Analytical methods to allow micro-sampling is under development at 'Núcleo de Desenvolvimento Farmacêutico e Cosméticos' (NUDFAC, Recife, Brazil).*

Whenever possible, the PK sample will be an aliquot from blood draws collected for other reasons such as safety lab.

All PK samples will be shipped to NUDFAC, Recife, Brazil for analysis. Drug concentrations will be measured by mass spectrometry.

8.5. Assessment of Safety

Safety and tolerability of benznidazole treatment will be assessed at Day 0 (post-dose), Day 7, Day 30, and Day 60. At each study visit, the patients will be questioned about current adverse events or any events observed during the period previous to the visit.

Also, patients will be advised to return to the clinic on any day during the follow-up period if they present any medical occurrence, as to allow for AE assessments at any unscheduled visits.

All AEs will be recorded in the appropriate section of the CRF (see details on Section 8.6).

8.5.1. Laboratory examinations

Laboratory parameters of Hematology and Chemistry will be measured at screening, Day 7, Day 30, Day 60, and in any unscheduled visit if clinically indicated. The volume of blood to be collected per visit is a total of 4mL: 1.5mL for Hematology and 2.5mL for Chemistries.

Safety lab will include: haemoglobin, total WBC count, differential WBC count, platelet count, ALT, AST, total, direct and indirect bilirubin, alkaline phosphatase and creatinine.

Normal lab parameters per age category are detailed in Appendix 1, as well as Common Terminology Criteria for Adverse Events (CTCAE v 3.0).

If any patient presents abnormal lab values, the clinician in charge will be responsible to evaluate if this finding is clinically significant or not. If the finding is considered clinically significant the abnormal lab value will be recorded as an adverse event in the appropriate CRF (see detailed information on session 8.6). Subsequent visits and follow-up of the adverse event will be at the discretion of the physician in charge of the patient.

8.6. Adverse event definitions and reporting

8.6.1. Adverse Event definition

An adverse event will be defined as any untoward medical occurrence (any unfavourable and unintended sign, symptom or disease, including an abnormal laboratory finding) in temporal association with the use of the investigational treatment and may or may not be causally related to it.

Abnormal laboratory (hematology and biochemistry) results will be reported as adverse events if the abnormality occurs or worsens after start of the study treatment, and if they are considered clinically significant by the Investigator, unless they are associated with an already reported clinical event.

The investigator or appropriate site personnel will examine any subject experiencing an AE as soon as possible. The investigator will do whatever is medically necessary for the safety and well being of the subject. The subject will remain under observation as long as a subject is receiving trial drug, and for 30 days following the last day of drug administration, or longer if medically indicated in the opinion of the investigator. All AEs observed or reported following administration of investigational treatment and felt to be related to the study treatment, will be followed until resolved or until medically stable.

All adverse events identified will be recorded in the appropriate AE section of the CRF using standard medical terminology in order to avoid the use of vague, ambiguous or colloquial expressions. Serious and/or unexpected adverse events will be notified by telephone, Email or fax to DNDi (see details on Section 8.6.5).

For the purposes of this trial, the PI of each site and DNDi will assume the responsibility of reporting SAEs to ANMAT, relevant Ethics Committees and other appropriate agencies, as needed, within the required timelines as per local legislation.

8.6.2. Serious Adverse Event

An adverse event will be defined as serious if it is

- fatal
- life-threatening
- requires or prolongs hospitalization
- results in persistent or significant disability
- is a congenital anomaly/birth defect
- results in an important medical event that may not be immediately life threatening or does not directly result in death or hospitalization, but which may jeopardize the patient or may require intervention to prevent the other outcomes listed above

8.6.3. Eliciting Adverse Event information

The investigator is required to report all directly observed adverse events and all adverse events spontaneously reported by the trial subject using concise medical terminology. In addition, during each study visit at Day 0, Day 7, Day 30 and Day 60 (end of treatment) subjects will be questioned about the occurrence of adverse events), with a generic question such as: Since last visit have you had any health problem?

8.6.4. Adverse Event reporting period

The adverse events reporting period for this trial begins

- Upon administration of the first dose of trial medication at Day 0 for non-serious events
- Upon subject enrolment in the trial (after signature of informed consent) for serious adverse events and ends at the last study visit (Day 60 visit).

All adverse events that occur during the adverse event reporting period specified above must be reported to DNDi, whether or not the event is considered related to the study medication. In addition, any adverse event that occurs subsequent to the adverse event reporting period that the investigator judges as possibly related to the investigational medication should also be reported as an adverse event.

8.6.5. Adverse Event and Serious Adverse Event reporting requirements

Information on AEs and SAEs must be evaluated by a study physician. Each adverse event is to be classified by the investigator as serious or non-serious. This classification will determine the reporting procedure for the event.

All AE/SAEs should be entered into the appropriate section of the CRF in a timely manner.

All serious adverse events (SAE) are to be reported immediately (within 24 hours of awareness of SAE by the investigator) to the DNDi medical coordinator, using the appropriate SAE report form. This includes a description of the event, onset date and type, duration, severity, relationship to study drug, outcome, measures taken and all other relevant clinical and laboratory data. The initial report is to be followed by submission of additional information (Follow-up SAE form) as it becomes available. Any follow-up reports should be submitted as soon as possible and if possible within 5 working days to the sponsor by fax, e-mail, and/or data acquisition system.

Serious adverse events should also be reported on the AE section of the case report form (CRF). It should be noted that the form for reporting of SAE (SAE form) is not the same as the adverse event section of the CRF. Where the same data are collected, the two forms must be completed in a consistent manner, and the same medical terminology should be used.

In addition to immediately reporting SAEs to DNDi, Investigators are responsible for reporting SAEs occurring at their site to their Independent Ethics Committee (IEC), and any periodic safety reporting, following the local requirements of their institution. The PI of each site will report the occurrence of SAE/unexpected AEs and regular periodic safety reports to ANMAT.

8.6.6. Grading of Adverse Event severity

Severity is a clinical determination of the intensity of an AE. The severity for an AE should be graded using the National Cancer Institute's Common Terminology Criteria

for Adverse Events (CTCAE, version 3.0). In case of AEs that are not described in the CTCAE v 3.0, the investigator will use the terminology MILD, MODERATE, or SEVERE to describe the maximum severity of the adverse event as follows:

MILD	Does not interfere with subject's usual functions
MODERATE	Interferes to some extent with subject's usual functions
SEVERE	Interferes significantly with subject's usual functions

This information on AE grading will be entered in the adverse event section of the CRF.

It is to be noted the distinction between severity and seriousness of adverse events. A severe adverse event is not necessarily a serious event.

8.6.7. Adverse Event causality assessment

For both serious and non-serious adverse events, the investigator is required to assess the possible relationship between the adverse event and the study drug, i.e. to determine whether or not there is a reasonable possibility that the study drug caused or contributed to the adverse event. To help investigators with the decision binary tree in the evaluation of causality, the CIOMS VI group recommends that investigators be asked to consider the following before reaching a decision:

- Medical history
- Lack of efficacy/worsening of existing condition
- Study medications
- Other medications (concomitant or previous)
- Withdrawal of study medication, especially following trial discontinuation / end of study medication
- Erroneous treatment with study medication (or concomitant)
- Protocol related procedure

The relationship of an AE to investigational treatment is assessed and determined by the investigator after careful consideration of the event in terms of biological plausibility, possible unrelated causes, any pre-existing medical conditions or concomitant medications, temporal relationship between administration of investigational treatment and the onset (or worsening) of the event, and known patterns of response to benznidazole in general.

Assessment of the relationship is based on the following guidelines:

Not Related: There is no temporal relationship to the investigational or control product or there is a plausible alternate explanation.

Related: All AEs are considered related if they are not judged as non-related and/or there is no compelling alternative aetiology.

The decision to suspend, and resume treatment or to permanently interrupt treatment due to an adverse event will be left to the PI.

8.6.8. Exposure *in utero*

Female subjects who have reached menarche will be excluded from this study. Therefore, it is not expected that exposure *in utero* will occur.

.

8.6.9. Adverse event follow up

All adverse events should be followed until they are resolved or the investigator assesses them as "chronic" or "stable" or the subject participation in the trial ends (*i.e*., until a final report is completed for that subject).

In addition, all serious adverse events and those non-serious events assessed by the investigator as possibly related to the investigational drug must continue to be followed even after the subject participation in the trial is over. Such events should be followed until they resolve or until the investigator assesses them as "chronic" or "stable." Resolution of such events is to be documented on the CRF.

9. Withdrawal and treatment discontinuation criteria

Patients will be considered to have completed the study if they satisfy all entry criteria, complete the course of treatment (a minimum of 50 days of treatment) and attend the Day 60 visit at the end of treatment.

Patients will be considered to have withdrawn from the study if they had entered into the study (*i.e.* gave informed consent and received at least one dose of treatment) but did not complete the treatment phase of the study and the final assessment at Day 60.

If a subject withdraws from the study, the reason must be noted on the CRF. If a subject is withdrawn from the study because of a treatment limiting adverse event, thorough efforts should be made to clearly document the outcome.

Treatment discontinuation does not imply in withdrawal from the study. In such cases, the treatment might be discontinued for a few days; therefore treatment will be incomplete or delayed. Benznidazole can be resumed, according to the assessment of the study investigator in charge of the patient. These patients should continue with study visits and assessments as planned, but the reasons for treatment discontinuation must be recorded in the appropriate source documentation and CRF.

If treatment is discontinued, PK sampling needs to be adjusted by drawing PK blood samples at least 3 days after treatment is resumed.

Reasons to indicate benznidazole treatment discontinuation will be described in the Patient Management SOP.

The following will be considered reasons to indicate patient withdrawal from the study:

- Severe skin reactions or repetitive moderate skin reactions
- Serious adverse events (despite relationship to study drug)
- Any condition that the investigators considers medically necessary to interrupt treatment and withdraw patient from the study
- Protocol violation
- Lost to follow-up

- Patient /caregiver / legal representative withdrawal of the consent
- Study termination by the Sponsor

If a subject does not return for a scheduled visit, every effort should be made to contact the subject. In any circumstance, every effort should be made to document subject outcome, if possible.

If the subject withdraws consent, no further evaluations should be performed and no attempts should be made to collect additional data, with the exception of safety data, which should be collected if possible.

Data obtained from withdrawn patients before his/her withdrawal will still be considered for PK analysis and safety analysis.

9.1. Rules for permanently interrupting study treatment

If a subject is withdrawn from the study before the full course of the treatment is completed, the physician must make all necessary arrangements to ensure that the subject receives the appropriate treatment for the relevant medical condition.

For patients who do not tolerate Benznidazole, rescue treatment will be provided with nifurtimox 10-15mg/Kg/day, divided in two-three daily doses for 60 days.

9.2. Subject withdrawal from the study and subject replacement

Subjects withdrawn from this study will not be replaced.

10. Data Analysis and Statistical Methods

10.1. Sample size determination

No formal sample size determination has been performed for this study. Sample size calculations for a given precision of the resulting PK parameter estimates require some prior knowledge regarding the variability in PK in the proposed study population. With the lack of PK data of benznidazole in children, the sample size was defined based on the expert's discussions and previous experience with medications with simple PK, the minimum size for population pharmacokinetics evaluation and logistical considerations.

PK estimates drove the sample size calculations. With the anticipated proportion of clinical failures of 5%, a sample size of 18 patients would suffice to demonstrate the prevalence of failures with 10% precision and 95% confidence level. An increased number of treatment discontinuations may occur in children with age >7 years-old due to the occurrence of adverse events. With an anticipated proportion of 10% clinical failures, a total of 35 children would be required to demonstrate the prevalence of failures with 10% precision and 95% confidence interval.

A minimum of 40 patients per age category was recommended by population PK experts to increase representativeness. Patient enrolment is to be stratified as follows:

- 1 day – 2 years: 40 patients to be enrolled, with a minimum of 10 newborns.
- 2 – 12 years: 40 patients.

10.2. Definition of study populations included in the analysis

For the population PK analysis, all subjects with any PK sampling will be included, as to have as many measurements as possible and representativeness of the study population.

For the efficacy analysis, intent-to-treat (ITT) and per protocol analysis will be conducted. However, it will only be possible to include subjects who present a positive PCR at screening and a second PCR assessment at the end of treatment (Day 60).

For the safety analysis, all subjects who have been exposed to the study drug will be included in the analysis.

10.3. Subject Disposition

At the end of the study it will be described:

- Number of patients who have been enrolled in the study (ITT set).
- Number (%) of patients who have received at least one dose of study drug (safety population)
- Number (%) of patients who have received a full course of treatment and completed the end of treatment (Day 60) assessments (per protocol set)
- Number (%) of patients who have completed the study.
- Number (%) of patients who have withdrawn from the study and reasons for withdrawal.
- Number (%) of subjects who completed the study visits at Days 0, 7, 30 and 60.
- Number (%) of subjects with at least one protocol violation and nature of protocol violation.

10.4. Baseline

Baseline characteristics of the study population will be described: age distribution, gender, positive parasitemia and parasite load at baseline and phase of disease development (acute vs. chronic).

10.5. Treatment Compliance

A full course of benznidazole treatment will be considered a minimum of 50 days of treatment.

At Day 0 and Day 30 the patient care-taker/legal representative will receive enough medication for the subsequent 30 days of treatment and a form (diary) for daily recording of information regarding dose administration. Also, they must bring all remaining study drugs on Day 30 and Day 60 visits to check for drug accountability.

The information on treatment compliance will be recorded in the appropriate section of the CRF.

10.6. Efficacy Analysis

The primary efficacy endpoint is the parasitological cure rate as determined by qualitative PCR at the end of treatment (Day 60).

Only patients with a positive PCR at screening and a PCR assessment at Day 60 will be included in this analysis.

The frequency of patients with a negative PCR at the end of treatment will be described for the ITT and per protocol populations.

In the ITT population, patients who do not have the final outcome assessment (PCR at Day 60) will be considered as treatment failures.

This analysis will be done for the entire study population and for the 2 age strata separately.

10.7. Safety Analysis

Safety endpoints for this study are the rate of SAEs and/or AEs leading to treatment discontinuation, and the rate and severity of AEs.

The primary assessment of safety will be based on the incidence and severity of adverse events (AE), notable abnormal vital signs, and laboratory values.

The safety population will be the patients who have received at least one dose of the study medication.

The number (%) of patients with SAE and/or AEs leading to treatment discontinuation will be described. A narrative for each of the SAEs will be developed detailing all aspects related to the medical event.

The number (%) of patients presenting at least one AE will be described. Subjects with multiple events with the same preferred terms will be counted once. The maximum severity grade for each preferred term and body system will be summarized. If multiple events with the same preferred terms are recorded for a subject, the event with the maximum grade will be included in the analysis.

The influence of demographic covariates will be assessed first by graphical visual inspection of the individual estimates of the pharmacokinetics parameters vs. the covariate plots. Potentially or known influential covariates will be incorporated sequentially into the pharmacokinetics model. The typical value of a given

parameter (e.g., CL) will be modeled as linearly dependent on each covariate (e.g. body weight, age, etc). Categorical covariates will be coded as indicator variable 0/1.

The efficacy and safety endpoints will be correlated with the estimated PK parameters. A full analysis plan will be prepared before initiation of the analysis.

10.8. Pharmacokinetics Analysis

Descriptive statistics (i.e. mean, median, sd, variation coefficient, etc) will be used to describe the analyzed variables.

The pharmacokinetics parameters to be estimated in the population will be Volume of distribution (V), clearance (C), absorption rate constant (Ka/F), elimination rate constant (Ke).

Population pharmacokinetics modelling will be performed using non-linear mixed-effects modelling as implemented in the software NONMEM® VI (NONMEM Users Guides, (1989-2006). Beal, S.L., Sheiner L.B., Boeckmann, A.J. (Eds.) Icon Development Solutions, Ellicott City, Maryland, USA). This program uses mixed (fixed and random)-effects non-linear regression to estimate population parameter means with inter-individual and intra-individual (i.e., residual) variability.

To determine the basic structural pharmacokinetic parameters, a stepwise procedure will be used to find the model that best fits benznidazole data in plasma.

The influence of demographic covariates will be assessed first by graphical visual inspection of the individual estimates of the pharmacokinetics parameters vs. the covariate plots. Potentially or known influential covariates will be incorporated sequentially into the pharmacokinetics model. The typical value of a given parameter (e.g., CL) will be modelled as linearly dependent on each covariate (e.g. body weight, age, etc). Categorical covariates will be coded as indicator variable 0/1.

Interindividual variations in pharmacokinetics parameters will be described using an exponential error model with normally – distributed interindividual random variability with mean zero and variance ω2. An exponential error model will be used to describe the intra-patient (residual) variability.

Parameter estimation and model selection: The data will be fitted using the first-order conditional method (FOCE INTER in NONMEM). Model selection will be based on the likelihood ratio test, pharmacokinetics parameters point estimates, and their respective confidence intervals, goodness-of-fit plots and visual predictive checks (VPC). A model will be considered as a statistically significant improvement over a previous model if it produces a decrease in objective function of >10.8 for one additional parameter (Chi-Square; P < 0.001).

Diagnostic plots will be done in R (R Development Core Team 2009. R: A language and environment for statistical computing. R Foundation for Statistical Computing, Vienna, Austria. ISBN 3-900051-07-0, URL http://www.R-project.org.)

11. Independent Data Safety Monitor

A Data Safety Monitor (DSM), who is independent of the investigator and sponsors, will be identified prior to study initiation. The DSM will monitor the study in order to ensure that harm is minimised and benefits maximised for the study subjects. The DSM will review safety data on a need basis, review all information related to the occurrence of SAEs and AEs leading to treatment discontinuation, and issue recommendations about the study.

12. Quality Assurance and Quality Control Procedures

12.1. Investigator’s file

The investigator must maintain adequate and accurate records to enable the conduct of the study to be fully documented and the study data to be subsequently verified. These documents include Investigator’s Site File, subject clinical source documents and screening / enrolment logs. The Investigator’s Site File will contain the protocol/protocol amendments, CRF and query forms, IEC and regulatory approval with correspondence, sample informed consent, drug accountability records, staff curriculum vitae and authorization forms and other appropriate documents/correspondence etc.

12.2. Case report forms (CRFs)

Data will be collected by laboratory technicians, medical doctors, clinical officers and nurses authorized by the investigator. It will be supervised by the Investigator and signed by the investigator or by an authorised staff member. Study-specific information will be entered into the Case Report Form (CRF). Data that are derived should be consistent with the source documents or the discrepancies should be explained. All CRF data should be anonymised, ie identified only by the study identification number.

The investigator at each trial site should ensure the accuracy, completeness, legibility, and timelines of all data reported to the sponsor in the CRFs and any other additional information that is required. The investigator is responsible for keeping all consent forms, screening forms, CRF and the completed subject identification code list in a secure location.

12.3. Source documents

The verification of the CRF data must be by direct inspection of source documents. Source documents include subject hospital/clinic records, physician's and nurse's notes, appointment book, original laboratory reports, ECG, EEG, X-ray, pathology and special assessment reports, signed informed consent forms, consultant letters, and subject screening and enrolment logs.

The investigator must maintain source documents such as laboratory and consultation reports, history and physical examination reports, etc., for possible review and/or audit by DNDi and/or Regulatory Authorities. The Investigator / designee will record the date of each subject’s visit together with a summary of their status and progress in the study.

12.4. Record Retention

The investigator must keep all essential documents until at least 2 years after the last

approval of a marketing application in an ICH region and until there are no pending or contemplated marketing applications in an ICH region or at least 2 years have elapsed since the formal discontinuation of clinical development of the investigational product. Study documents should be retained for a longer period; however, if required by the applicable regulatory requirements or by an agreement with DNDi. It is the responsibility of the sponsor to inform the investigator/institution as to when these documents no longer need to be retrained. After that these documents may be destroyed with prior permission from DNDi, subject to local regulations.

Should the investigator wish to assign the study records to another party or move them to another location, DNDi must be notified in advance.

12.5. Monitoring, audits and inspections

Clinical Monitors will conduct regular monitoring visits, during which he/she will inspect and source data verify the Informed Consent Forms, medical records, laboratory results, imaging assessments, Case Report Forms, drug dispensing logs, and protocol violations.

Monitoring visits to the trial site will be made periodically by DNDi representatives or designated clinical monitors to ensure that GCPs and all aspects of the protocol are followed. Source documents will be reviewed for verification of consistency with data on CRFs. The investigator will ensure direct access to source documents by DNDi or designated representatives. It is important that the investigators and their relevant personnel are available during the monitoring visits.

The investigators will permit representatives of DNDi and/or designated clinical monitors to inspect all CRFs, medical records, laboratory work sheets and to assess the status of drug storage, dispensing and retrieval at anytime during the study. The corresponding source documents for each subject will be made available provided that subject confidentiality is maintained in accord with local regulations. The inspections are for the purpose of verifying the adherence to the protocol and to ensure the study is conducted according to GCP. It is important that the investigators and other trial site staff are available at these visits.

The monitoring visits provide DNDi with the opportunity to evaluate the progress of the study, verify the accuracy and completeness of CRFs, resolve any inconsistencies in the study records, as well as to ensure that all protocol requirements, applicable regulations, and investigator's obligations are being fulfilled. Four visit types are planned: pre-study, study start, during the study, and study end. Visits may also be performed by regulatory authorities.

It will be the clinical monitor’s responsibility to inspect the CRF at regular intervals throughout the study, to verify the adherence to the protocol and the completeness, consistency and accuracy of the data being entered on them. The investigator agrees to cooperate with the clinical monitor to ensure that any problems detected in the course of these monitoring visits are resolved.

12.6. Audits and inspections

The trial site may also be subject to quality assurance audits by DNDi or designated representatives and/or to inspection by regulatory authorities or Independent Ethics Committees (IEC).

It is important that the investigators and their relevant personnel are available for possible audits or inspections.

12.7. Data Management

After the CRF has been completed and monitored by the clinical monitor, CRFs will be collected and data will be entered onto a database using double independent data entry. The trial data will be stored in a computer database maintaining confidentiality in accordance with national data legislation.

In order to ensure data quality, a uniform hard copy CRF will be designed for use at all the sites. Data will then be sent to the Data centre or to CRO for data entry and data cleaning.

12.8. Confidentiality of trial documents and subjects records

The investigator must assure that subjects' anonymity will be maintained and that their identities are protected from unauthorized parties. On CRFs or other documents submitted to the sponsor, subjects should not be identified by their names, but exclusively by an identification code. The investigator should keep a subject enrolment list showing codes, names, and addresses. The investigator should maintain documents for submission to sponsor authorized representative, and subject's signed written consent forms, in strict confidence.

13. Protocol Amendments

The Principal investigator will ensure that the study protocol is strictly adhered to throughout, and that all data are collected and recorded correctly on the CRF. The Principal investigator may contact the medical coordinator for a protocol waiver for minor deviations from the protocol, e.g. patient unable to attend during visit window.

All protocol modifications must be documented in writing. Any protocol amendment must be approved and signed by the sponsor and the Principal investigator and is to be submitted to the appropriate IEC for information and approval in accordance with local requirements, and to regulatory agencies if required. Approval by IEC (and Regulatory Authority, if applicable) must be awaited before any changes can be implemented, except for changes necessary to eliminate an immediate hazard to trial subjects, or when the change involves only logistical or administrative aspects of the trial [e.g. change in clinical monitor[s], change of telephone number[s].

The protocol amendment can be initiated by either sponsor or by any Principal investigator.

The investigator will provide in writing the reasons for the proposed amendment and will discuss with the medical coordinator and sponsor.

14. Termination of the Study

Both the sponsor and the investigator reserve the right to terminate the study at any time prior to inclusion of the intended number of subjects, but they intend to exercise this right only for valid scientific or administrative reasons. Should this be necessary, both parties will arrange the procedures on an individual study basis after review and consultation. In terminating the study, the sponsor and the investigator will assure that adequate consideration is given to the protection of the subject's interest.

Reasons for termination by the sponsor(s) may include but not be limited too:

- Too low enrolment rate.
- Protocol violations.
- Inaccurate or incomplete data.
- Unsafe or unethical practices.
- Questionable safety of the test article.
- Suspected lack of efficacy of the test article.
- Following the recommendation of the DSM or IEC
- Administrative decision.

Reasons for termination by the investigator may be:

- Insufficient time or resource to conduct the study
- Lack of eligible patients

In the event that a study is terminated either by the sponsor or by the investigator, the investigator has to:

- Complete all CRFs to the greater extent possible
- Return all test articles, CRF, and related study materials to the sponsor who provided them
- Answer all questions of the sponsors or their representatives related to data of subjects enrolled at the site prior to study termination
- Ensure that subjects enrolled in the study who had not yet reached a follow up time point are followed up with the necessary medical care.
- Provide in writing the reasons for his decision to the national health authority and the sponsor.

15. Ethics

The experimental protocol for this study has been designed in accordance with the general ethical principles outlined in the Declaration of Helsinki and ICH guidelines for Good Clinical Practice (International Committee for Harmonization). DNDi assures that it will comply with all applicable state, local and foreign laws for protecting the rights and welfare of human subjects. This protocol and any protocol amendments will be reviewed / approved by an IEC before its implementation.

It is the responsibility of the Investigator to apply for review to the IEC of the country

where the study takes place regarding local rules and regulations. Written approval from all involved IECs must be obtained before implementation of any protocol-specified intervention /investigation provided to the subject [such as subject information sheets or descriptions of the study].

Any modifications made to the protocol after receipt of the IEC approval must also be submitted by the investigator in writing to the IEC in accordance with local procedures and regulatory requirements.

15.1. Informed consent process

Inclusion in the study will occur only if the parent/guardian (for all children) gives written informed consent. It is the responsibility of the investigator / designee to obtain written informed consent from each individual participating in this study, after adequate presentation of aims, methods, anticipated benefits, and potential hazards of the study. The written informed consent document will be translated into the local language or a language understood by the subject(s). If needed, the person will be given time to discuss the information received with members of the community or family before deciding to consent. The subject or parent/guardian will be asked to provide written and signed consent.

If the subject is illiterate, a literate witness must sign (this person should have no connection to the research team, and, if possible, should be selected by the participant).

The investigator should also obtain the assent of children (if > 7years), but their assent must be completed by the permission of a parent or guardian.

If new safety information results in significant changes in the risk/benefit assessment, the consent form should be reviewed and updated if necessary. All subjects (including those already being treated) should be informed of the new information, given a copy of the revised form and give their consent to continue in the study.

15.2. Ethical aspects of subject inclusion and study procedures

Participation in this study will imply only minimal risk (blood collection for filter paper with needle) on routine care in the management of children with Chagas Disease.

15.3. Ethical aspects of study treatments

Benznidazole is the standard of care for Chagas disease treatment.

15.4. Patient costs

Patients will be reimbursed for travel to and from the study site but will not receive any payment for trial participation. The payments of the lost profits for parents/legal representative will be considered in case it is applicable.. Any medication that is required for the treatment of CD during the trial period will be provided free of charge

to the patient. Food during the in-patient treatment phase will also be provided free of charge to the patient. This is seen as an essential part of the patient care plan bearing in mind the high prevalence of malnutrition and the poverty of these patients.

16. Insurance and Liability

DNDi will provide insurance against claims arising from the trial, except for claims that arise from malpractice and/or negligence.

In addition, the DNDi will address the costs of treatment of trial subjects in the event of trial-related injuries in accordance with the applicable regulatory requirement.

17. Reporting and publication

All clinical trials will be registered with a recognised clinical trial registry such as www.clinicaltrials.gov.

18. References

Andrade A.L., Martelli C.M., Oliveira R.M., Silva S.A., Aires A.I., Soussumi L.M., Covas D.T., Silva L.S., Andrade J.G., Travassos L.R., Almeida I.C. Short report: benznidazole efficacy among Trypanosoma cruzi-infected adolescents after a six-year follow-up. *Am J Trop Med Hyg* 2004; 71(5): p. 594-7.

de Andrade A.L., Zicker F., de Oliveira R.M., Almeida Silva S., Luquetti A., Travassos L.R., Almeida I.C., de Andrade S.S., de Andrade J.G., Martelli C.M. Randomized trial of efficacy of benznidazole in treatment of early Trypanosoma cruzi infection. *Lancet* 1996; 348:1407–1413.

Carlier Y. & Torrico F. Congenital infection with *Trypanosoma cruzi*: from mechanisms of transmission to strategies for diagnosis and control. *Rev Soc Bras Med Trop* 2003; 36(6):767-71.

Food and Drug Administration (FDA). Guidance for Industry: General Considerations for Pediatric Pharmacokinetic Studies for Drugs and Biological Products , Draft Guidance. 1996, http://www.fda.gov/downloads/Drugs/GuidanceComplianceRegulatoryInformation/Guidances/UCM072114.pdf

Food and Drug Administration (FDA). Guidance for Industry: Population Pharmacokinetics.1999, http://www.fda.gov/downloads/Drugs/GuidanceComplianceRegulatoryInformation/Guidances/UCM072137.pdf

Garcia-Bournissen F, Altcheh J, Giglio N, Mastrantonio G, Della Védova CO, Koren G. Pediatric clinical pharmacology studies in Chagas disease: focus on Argentina. *Paediatr Drugs* 2009; 11(1):33-7.

Hotez, P.J, Bottazzi, M.E., Franco-Paredes, C., Ault, S.K., and Periago, M.R. The neglected tropical diseases of Latin America and the Caribbean: a review of disease burden and distribution and a roadmap for control and elimination. *PLOS Negl. Trop. Dis.* 2008; 2(9):e300.

Kirchhoff LV. Changing Epidemiology and Approaches to Therapy for Chagas Disease. *Curr Infect Dis Rep*. 2003; 5(1):59-65.

Mathers, C.D., A. Lopez, and C.J.L. Murray. The Burden of Disease and Mortality by Condition: Data, Methods, and Results for the Year 2001. Global Burden of Disease and Risk Factors, ed. A. Lopez, *et al*. 2006, New York: Oxford University Press.

Ministerio de Salud de la Nación, Argentina. Centro Nacional de Diagnóstico e Investigación de Endemoepidemias CENIDE. Guia de Atención a la Enfermedad de Chagas. 2005.

Pan American Health Organization. Estimación Cuantitativa de la Enfermedad de Chagas en las Americas. 2006. http://www.bvsops.org.uy/pdf/chagas19.pdf

Pereira KS, Schmidt FL, Guaraldo AM, Franco RM, Dias VL, Passos LA. Chagas' disease as a foodborne illness. *J Food Prot*. 2009; 72(2):441-6.

Pinto Dias, J.C. The treatment of Chagas disease (South American trypanosomiasis). *Ann Intern Med* 2006; 144(10): p. 772-4.

Rodriques Coura, J. and S.L. de Castro, A critical review on Chagas disease chemotherapy. *Mem Inst Oswaldo Cruz* 2002; 97(1): p. 3-24.

Raaflaub J, Ziegler WH. Single-dose pharmacokinetics of the trypanosomicide benznidazole in man. *Arzneimittelforschung* 1979; 29(10):1611-1614.

Raaflaub J. Multiple-dose kinetics of the trypanosomicide benznidazole in man. *Arzneimittelforschung* 1980; 30(12):2192-2194.

Ribeiro I., Sevcsik A.M., Alves F., Diap G., Don R., Harhay M.O., Chang S., Pecoul B. New, improved treatments for Chagas disease: from the R&D pipeline to the patients. *PLoS Negl Trop Dis*. 2009; 3(7):e484.

Schijman AG, Altcheh J, Burgos JM, Biancardi M, Bisio M, Levin MJ, Freilij H. Aetiological treatment of congenital Chagas' disease diagnosed and monitored by the polymerase chain reaction. *J Antimicrob Chemother* 2003; 52(3):441-9.

Schmunis, G., *A tripanossomiase Americana e seu impacto na saude publica das Americas.* In *Trypanosoma cruzi e doença de Chagas,* 2nd ed., 2000: p. 1-15.

Schmunis, G.A. Epidemiology of Chagas disease in non-endemic countries: the role of international migration. *Mem Inst Oswaldo Cruz* 2007; 102, Suppl 1: 75-85.

Sosa-Estani, S. and E.L. Segura, *Etiological treatment in patients infected by*

Trypanosoma cruzi: experiences in Argentina. Curr Opin Infect Dis 2006; 19(6): 583-7.

Sosa Estani S., Segura E.L., Ruiz A.M., Velazquez E., Porcel B.M., Yampotis C.. Efficacy of chemotherapy with benznidazole in children in the indeterminate phase of Chagas' disease. *Am J Trop Med Hyg.* 1998; 59(4):526-9.

World Health Organization (WHO 1991. *OMS Technical Report Series No. 811.*

World Health Organization (WHO) 2002. Control of Chagas Disease, Second report of the OMS Expert Committee. *Technical Report Series No* 905.

WorldBank, *World Development Report 1993: Investing in Health*. 1993, New York: Oxford University Press.

Yadon, Z. E. & Schmunis, G.A. Congenital Chagas Disease: Estimating the Potential Risk in the United States. *Am. J. Trop. Med. Hyg*. 2009; 81(6): 927-933.

Appendices

CTCAE v 3.0

Normal lab parameters
